# SUMO modification in apoptosis

**DOI:** 10.1007/s10735-020-09924-2

**Published:** 2020-11-22

**Authors:** Peiyao Li, Huiru Jing, Yanzhe Wang, Lei Yuan, Hui Xiao, Qian Zheng

**Affiliations:** Key Laboratory of the Ministry of Education for Medicinal Plant Resources and Natural Pharmaceutical Chemistry, National Engineering Laboratory for Resource Development of Endangered Crude Drugs in the Northwest of China, College of Life Sciences, Shaanxi Normal University, Xi’an, 710119 China

**Keywords:** Apoptosis, Apoptotic cell clearance, SUMO, Senps

## Abstract

Apoptosis and clearance of dead cells is highly evolutionarily conserved from nematode to humans, which is crucial to the growth and development of multicellular organism. Fail to remove apoptotic cells often lead to homeostasis imbalance, fatal autoimmune diseases, and neurodegenerative diseases. Small ubiquitin-related modifiers (SUMOs) modification is a post-translational modification of ubiquitin proteins mediated by the sentrin-specific proteases (SENPs) family. SUMO modification is widely involved in many cellular biological process, and abnormal SUMO modification is also closely related to many major human diseases. Recent researches have revealed that SUMO modification event occurs during apoptosis and clearance of apoptotic cells, and plays an important role in the regulation of apoptotic signaling pathways. This review summarizes some recent progress in the revelation of regulatory mechanisms of these pathways and provides some potential researching hotpots of the SUMO modification regulation to apoptosis.

## Introduction

Apoptosis is an evolutionarily conserved cell progress from nematode to mammals, which is of great importance for organisms normal development, failure to apoptotic cells clearance can result in the release of toxins and inflammation of the surrounding environment (Elliott and Ravichandran 2010; Munoz et al. 2005). *Caenorhabditis elegans* is an important genetic system for studying the phagocytic mechanism of apoptotic cells, and the molecular mechanism of apoptotic cell clearance has been extensively studied in *C. elegans* (Reddien and Horvitz 2004; Lettre and Hengartner 2006). Two major signaling pathways trigger apoptotic cell death: the mitochondrial (internal) pathway and the death receptor (external) pathway. Normally, apoptotic cells are recognized and degraded by phagocytes (such as macrophages) before releasing potentially toxic substances (Galluzzi et al. 2012). Defective apoptotic cell clearance can lead to a variety of diseases, including autoimmune diseases, neurodegenerative disorders.

Small ubiquitin-related modifier (SUMO) is involved in diverse cellular processes and disorder of sumoylation is implicated in the pathogenesis of human diseases. Proteins are the physical basis of life and the basic unit of the function and structure of organisms. The Small Ubiquitin-like Modifier (SUMO) post-translational modify a large number of proteins that function in diverse biological processes, including transcription, chromatin remodeling, DNA repair, and mitochondrial dynamics. SUMOylation is also a dynamic process, which can be reversed by a family of conserved Sentrin/SUMO-specific proteases (SENPs). Post-translational modification of ubiquitin-like proteins has been shown to be involved in many regulatory processes of cellular activities, such as cell cycle, signaling, immune recognition, apoptosis, cell proliferation and differentiation, inflammation, and DNA damage repair (Gareau and Lima 2010; Zhao et al. 2004; Muller et al. 2001).

In this review, we briefly describe the process of cell death and the SUMOylation then discuss the relationship between apoptosis and ubiquitination-like modification.

## Mechanisms of apoptosis

Lockshin and Williams originally defined programmed cell death in the context of insect development. Subsequently, Kerr et al. noticed by ultrastructural analysis two morphologically different types of cell death in humans, apoptosis, and necrosis (Lockshin and Williams 1965; Kerr 1971).

During apoptosis, the cells shrink with integral but ruffling plasma membranes, and nuclei are condensed and fragmented (Saraste and Pulkki 2000). Apoptosis is one of the important ways of programmed cell death, the process can be divided into several important steps, including the initiation and transmission of death signals, the triggering of death procedures, and the clearance of apoptotic cells (Elmore 2007). Apoptosis is mediated by a series of caspases that function in the cascades, and caspase 3 or caspase 7 is responsible for killing cells at the end of caspase 3 or caspase 7. Apoptosis involves not only the mechanism of killing cells, but also the release of “find me” signals to recruit macrophages and the exposure of “eat me” signals to bind macrophages, which efficiently promotes the elimination of apoptotic cells (deCathelineau and Henson 2003; Medina and Ravichandran 2016).

Apoptotic cells are cleared by specialized phagocytes, such as macrophages and dendritic cells, or by non-specialized phagocytes, such as epithelial cells. This process plays a very important role in organ formation and tissue dynamic balance (Arandjelovic and Ravichandran 2015). Apoptotic cells are swiftly phagocytosed by macrophages to prevent the release of intracellular components, timely and effective elimination of apoptotic cells can also prevent the body from producing inflammation or autoimmune diseases. In contrast, failure to remove the apoptotic cell often leads to a variety of severe chronic inflammatory or fatal autoimmune diseases (systemic lupus erythematosus), as well as neurodegenerative diseases (Alzheimer's disease) (Wang and Yang 2016; Nagata et al. 2010).

Apoptosis can be defined as cell death accompanied by activation of the caspase protease. Apoptosis is strictly controlled by multiple factors, stages, and genes, which is regulated by the following signal transduction pathways: mitochondrial pathway, death receptor signaling pathway, and so on.

Apoptosis involves the activation of caspases, which can destroy all morphological changes of cell death. Caspases are cysteine proteases that are specific to aspartic acid residues in the substrate. Although there are at least 17 different caspases in mammals, we focus on only a small number of them, and the activation of these caspases is at least partially understood, and the role of these caspases in cell death has been determined.

## The mitochondrial pathway of apoptosis

When cells are regulated by internal apoptotic stimulators (Oncogene activation, DNA damage, hypoxia, growth factor damage, endoplasmic reticulum stress, etc.), the mitochondrial pathway, an intrinsic pathway of cell apoptosis, is activated and thus cell apoptosis is triggered. This process increases mitochondrial membrane permeability and releases Cyt-C into the cytoplasm to interact with Apaf1, leading to a series of Caspase cascade reactions.

The mitochondrial pathway of apoptosis, also known as the intracellular pathways of apoptosis, is triggered by internal apoptotic stimulators (Oncogene activation, DNA damage, hypoxia, growth factor damage, endoplasmic reticulum stress, etc.) (Bratton and Salvesen 2010; Bratton et al. 2001). During apoptosis, cytochrome c is released from the mitochondria into the cytoplasm and binds to apoptotic protease activator 1 (APAF1, CED-4 in *C.elegans*), which triggers the hydrolysis of the Apaf1 cofactor dATP to dADP (Adrain et al. 1999). The subsequent exchange of dADP and exogenous dATP resulted in the aggregation of 7 apaf1-datp-cyto-chromeon-c cells into an active apoptotic body (Kim et al. 2006). At the apoptotic corpuscle center, APAF1 activates the inactive caspase 9 monomer (Thornberry and Lazebnik 1998). In healthy cells, cytochrome c is present only in the mitochondrial membrane gap. In addition to cytochrome c, two other pro-apoptotic proteins are released during this process: Smac (also known as diablo) and Omi(also known as HtrA2) (Thornberry and Lazebnik 1998; Zou et al. 1999). MOMP is an event strictly controlled by members of the Bcl2 family. There are three main types of Bcl2 proteins: pro-apoptotic effectors ( Bax and Bak); Anti-apoptotic Bcl2 proteins (such as Bcl2, Bcl-xl, Mcl1); And bh3-specific proteins (activating pro-apoptotic effector factor and neutralizing anti-apoptotic Bcl2 protein). CED-9 protein in *C.elegans* is similar to human anti-apoptotic protein Bcl2/ Bcl-xl. And EGL-1 is similar to several bh3-specific proteins. In the embryo of *C. elegans*, CED-9 and CED-4 are located in the mitochondria, and CED-4 is isolated in the mitochondrial membrane. When cell apoptosis occurs, *egl-1* is activated by transcription and interacts with CED-9 to convert CED-4 from the mitochondrial surface to the peri-nuclear region, thus promoting the activation of CED-3 caspase of cells and eventually leading to cell death (Tait and Green 2010; Tait et al. 2010; Yuan and Horvitz 1990; Yan et al. 2005).

The CED-3 caspase, a homologous gene of human CASP2 (caspase 2), is involved in the positive regulation of apoptosis. Studies have shown that Dicer/dcr-1 ribonuclease is a substrate of CED-3. In the process of apoptosis, CED-3 can convert DCR-1 into deoxyribonuclease and initiate chromosome breakage. Moreover, mitochondrial fission proteins drp-1/Drp1 and multi-transmembrane protein CED-8/Xk1 also undergo CED-3 cleavage, and the cleaved DRP-1 and CED-8 promote the exposure of phosphatidylserine (PtdSer) as a "eat me" signal on the surface of apoptotic cells (Nakagawa, et al. 2010; Chen et al. 2013a; Breckenridge et al. 2008).

## Death receptor signaling pathway

The death receptor (DRs) pathway is one of the important pathways mediating apoptosis and can trigger apoptosis. DRs belongs to tumor necrosis factor receptor (TNFR) and has an extracellular domain rich in Cys and an intracellular death domain (DD). When the death receptor binds to a specific death ligand, the extracellular death signal activates the intracellular apoptosis mechanism through DRs, inducing apoptosis. The known death receptors-ligands are Fas (APO-1, CD95)-FasL (CD95L), TNFR1-TNF, TRAILR1/2-TRAIL. (I) In the process of Fas-FasL signal transduction, FADD, Daxx, FILP, and other related proteins are recruited by intracellular terminal DD; (II) after TNFR1-TNF binding, THE TNFR1 DD is induced to aggregate and recruit the bridging protein TRADD, which can recruit signal molecules such as TRAF2, RIP and FADD; (III) TRAILR1/2-TRAIL binds to FADD in cancer cells via DD. FADD then recruits pro-caspase8 through the death effect domain DED to form the death-induced signaling complex (DISC), and the pro-caspase8 on the DISC cuts itself into the active caspase8, thus mediating apoptosis (Carrington et al. 2006).

In type I cells, activated caspase-8 promotes apoptosis by dividing and activating caspase-3 and caspase-7. In type II cells, XIAP inhibits active caspases, thereby preventing apoptosis. Caspase-8 mediated cleavage activates Bid, which in turn activates Bax and Bak to promote MOMP. IAP antagonists Smac and Omi were subsequently reused to neutralize XIAP and allow apoptosis to continue. The signaling pathway downstream of TNFR1 is more complex and may lead to three different outcomes: survival, apoptosis, or necrosis. TNFR1-associated death domain protein (TRADD) does not directly activate caspase-8, but it can serve as a membrane-binding scaffold for additional signaling molecules, including kinases, receptor interaction protein 1 (RIP1), and ubiquitin-ligases TRAF2 and cIAP1/2. When the ubiquitination of RIP1 is inhibited by the ubiquitin ligase contained in complex I (such as cIAP1/2) or directly deubiquitinated by enzymes such as CYLD, TNFR1 ligation can lead to cell death. RIP1 and TRADD are released from TNFR1 to form a series of dynamic cytosolic signaling platforms (collectively, complex II) that may lead to cell apoptosis and necrosis. Cytoplasmic TRADD recruits FADD (via dd-dd interaction) and activates caspase-8. In this structure, the formation of complex II leads to apoptosis and death (Newton et al. 2012; Jost et al. 2009).

## Apoptotic cell recognition

The most-studied ‘eat-me signal’ is phosphatidylserine (PS), a phospholipid that is exposed on the surface of dying cells (Leventis and Grinstein 2010). In normal cells, PS is mainly located in the inner leaflet of the cell membrane, and it is continuously transferred from the outer lobule to the inner lobule of the cell membrane by amino-phosphatidyltransferase TAT-1, to maintain its asymmetric distribution on the cell membrane (Fadok et al. 1992; Williamson and Schlegel 2003). Not only PS and calreticulin, an intracellular chaperone protein involved in the transport of correctly folded proteins through the endoplasmic reticulum, may become exposed on the surface of apoptotic cells (Darland-Ransom et al. 2008; Kuraishi et al. 2007). In *C.elegans,* TTR-52 is an extracellular protein similar to trans-thymosin secreted by non-apoptotic intestinal cells, which can recognize and bind to the exposed PS on the surface of apoptotic cells. TTR-52 promotes the movement of PS vesicles from apoptotic cells to phagocytes together with the ABC transporter protein, CED-7 (Mapes et al. 2012). Meanwhile, TTR-52 interacts with the extracellular domain of phagocytic receptor CED-1, consequently delivers the “eat me” signal to phagocytes, accomplishing the clearance of apoptotic cells (Zhou et al. 2001). The CED-1 homolog in *Drosophila*, Draper (Drpr) has also been shown to play a role in the elimination of apoptotic cells by hemocytes/macrophage both in vitro and in vivo (Manaka et al. 2004). In addition to the phagocytic receptor CED-1, the phosphatidylserine receptor *psr-1*, an evolution-conserved PtdSer receptor, also mediates cell corpse recognition by PS, and then promotes the elimination of apoptotic cells through CED-2, CED-5, and CED-12 signaling pathways (Wang et al. 2003).

## Cell corpse clearance

The clearance of apoptotic cells involves several dynamic steps including: mutual recognition between apoptotic cells and the phagocytic receptors, cytoskeleton rearrangement in phagocytes, phagosome formation after endocytosis, and the ultimate degradation of apoptotic cells in phagocytes (Li et al. 2013). Apoptotic cell clearance is highly evolutionarily conserved, but this process is extremely redundant and complex in mammalian systems, with multiple molecules playing a similar role in apoptotic cell clearance (Wang et al. 2003). By studying the genetics of programmed cell death in model animal, the molecular mechanism of apoptotic cells clearance has been preliminarily understood.

## Phagocytic cytoskeletal rearrangement and phagosome sealing

On the one hand, CED-1 interacts with CED-6 to recruit clathrin CHC-1 and the multi-subunit adaptor protein AP2, forming a complex to mediate actin cytoskeleton rearrangement (Chen et al. 2013b); on the other hand, AP2 also interact with LST-4 and DYN-1 to initiate the cell intima into the "phagocytic cup", subsequently causes cytoskeletal rearrangement and promotes the extension of phagocytic pseudopodia (Yu et al. 2006). Another pathway that controls the phagocytosis of apoptotic cells by adjacent cells is composed of CED-2, CED-5, CED-10, CED-12, and PSR-1 (Hsu and Wu 2010). PSR-1 is involved in the recognition and binding of PtdSer to initiate apoptosis cell clearance. Non-receptor tyrosine kinase SRC-1 can combine with INA-1 and CED-2 to transmit phagocytosis signal to the downstream. CED-2 interacts with CED-5, and CED-5 can form a complex with CED-12 to activate CED-10 as a guanosine exchange factor. CED-10 is a homologous of human RacI guanosylates hydrolase, which can promote the rearrangement of the cytoskeleton and the phagocytosis of apoptotic cells (Liu and Hengartner 1999).

Under the action of these two pathways, phagocytes of *C. elegans* continuously extended to completely envelop apoptotic cells in the membrane of phagocytic cells, and finally form phagocytes. Phagocytic pseudopodia fusion completely encloses the phagocytic body of apoptotic cells. PtdIns(4,5)P2 and PtdIns3P phosphoinositol play a very important role in the closure of phagocytes. PtdIns(4,5)P2 is catalyzed by PtdIns4P 5 kinase ppk-1 and is mainly concentrated in the unsealed phagocytes. PtdIns3P phosphatase MTM-1 (myotubularin) and PIKI-1 of type II phosphoinositol 3 kinase synergistically controlled the content of PtdIns3P in unsealed phagocytes. Then, the SNX9 family protein LST-4 was recruited to the phagocytosis by detecting PtdIns(4,5), P2, PtdIns3P, MTM-1, and LST-4 further recruited DYN-1 to complete phagocytosis (Cheng et al. 2015).

## Phagosome formation and maturation

Phagocytic maturation includes the processes from the generation of phagocytes to the fusion of lysosomes and phagocytes (Vieira et al. 2002). This process starts immediately after the phagocytic sealing, even before this. After the phagosome is sealing, it falls off from the cell membrane, and fuses with the early endosome, late endosome, and lysosome successively, and fuses with the lysosome to form phagolysosome. At this time, various hydrolytic enzymes in the lysosome form an acidic environment to degrade the phagosome. It has been found that Rab GTPases (Rab1/2/3/4/5/7/11/14) play a very important role in the fusion of phagocytes and lysosomes and the acidification of phagolysosomes, such as regulating the clearance of apoptotic cells and phagocytosis (Stuart et al. 2007; Garin et al. 2001). It has been shown that early phagocytosis recruited RAB-5 protein in mammals, *Drosophila*, and *C. elegans*, while RAB-7 participates in the late stage of phagosome maturation and mediates phagocytic and lysosomal fusion (Vieira et al. 2002; Kinchen and Ravichandran 2008; Kinchen et al. 2008).

RAB-5 binds to early phagocytes, which may promote the production of PtdIns3P by activating type III phosphoinositol 3 kinase VPS-34 (Kinchen et al. 2008). Effector molecules of PtdIns3P, such as SNX-1 and SNX-6, are recru ited into the phagolysosome and mediate the recycling of the phagocytic receptor CED-1 (Norris et al. 2017). Then, with the help of SAND-1, CCZ-1, and GTPase activating protein (GAP) TBC-2, the phagocytes undergo a gradual maturation phase that exchanges the combination from RAB-5 to RAB-7 through the Rab exchange mechanism (Li et al. 2009; Kinchen and Ravichandran 2010). The other two RAB GTPases, RAB-14 and UNC-108(the RAB-2 homolog of *C.elegans*), and the activated molecule gopher 1 of UNC-108 play a role in the maturation of phagocytes earlier than RAB-7. Rab-14 and UNC-108 are likely to facilitate the recruitment of lysosomes to phagocytes, while RAB-7 acts as an anchor to fuse lysosomes and phagocytes. In addition, the HOPS complex and the small GTPase ARL-8 are also essential for the formation of phagocytic lysosomes. Finally, the lysosomal lysine/arginine transporter LAAT-1 maintains the lysosome formation, the lysosomal cathepsin L(CPL-1), and type II DNA enzyme (NUC-1) mediate the digestion and degradation of apoptotic cells (Liu et al. 2012; Xu et al. 2014).

## Synopsis of SUMO conjugation and deconjugation

SUMOylation, similar to ubiquitination, can covalently modify lysine residues of target proteins and is an important way of reversible post-translational modification of target proteins. It has been found that SUMOylation has biological significance for the function, localization, and stabilization of target proteins, thus affecting cell growth, differentiation, and apoptosis. Single/poly SUMOylation involves many biological processes, such as regulation of gene transcription, protein–protein interactions, nucleoplasmic transport, signal transduction, and protein degradation. In addition to the involvement of sumoylation in the regulation of protein–protein and protein-DNA interactions, the most important thing is that more studies have found that SUMOylation of proteins may be related to the occurrence and development of human diseases, such as cancer, neurodegenerative diseases, and immune diseases (Flotho and Melchior 2013; Nie and Boddy 2016).

## SUMO proteins

SUMO is an 11-kDa protein that is a highly conserved family and widely found in eukaryotes. Yeast, nematode, and fruit fly have only one SUMO gene, while the human genome encodes four different SUMO proteins: SUMO1-SUMO4 (Table 1). SUMO1-SUMO3 are widely expressed, while SUMO4 is only expressed in specific tissues, such as kidney and spleen (Johnson 2004). Moreover, the mature SUMO2 and SUMO3 share 97% sequence identity to form poly-SUMO chains, while their homology with SUMO1 is only 50% (Johnson 2004). SUMO1 is very different from SUMO2/3 in function, and previous data have shown that they have distinct and overlapping sets of target proteins and the substrate protein it binds to in the body is also different (Saitoh and Hinchey 2000; Tatham et al. 2001; Vertegaal et al. 2006).

**Table 1:** List of currently identified SUMO related proteins in *Homo sapiens*, *Mus musculus*, *Caenorhabditis elegans* and *Drosophila melanogaster*, giving all their known names

Protein type	*Homo sapiens*	*Mus musculus*	*Caenorhabditis elegans*	*Drosophila melanogaster*
Modifier	SUMO1 SUMO2/3 SUMO4	SUMO1 SUMO2/3	SMO-1(CeSUMO)	Smt3
E1	SAE1	Aos1	AOS-1	SAE1
Activating enzyme	SAE2	Uba2	UBA-2	SAE2
E2 Conjugating enzyme	UBC9	Ubc9	UBC-9	Ubc9
E3 Ligases	PIAS1	PIASs	GEI-17	Su(var)
	PIAS2	RanBP2	MMS-21	2–10
	PIAS3			
	PIAS4			
	NSE2			

The same substrate protein can be modified by different subtypes of SUMO protein, and even modified by the same subtype of SUMO protein can lead to different biological effects due to the various stimulus factors. SUMO family is conjugated to proteins which regulate a variety of cellular processes such as nuclear transport, transcription, chromosome segregation, and DNA repair, suggesting that SUMO signaling plays important roles in diverse processes by altering proteins activities, their ability to interact with other proteins, or their subcellular localization (Dohmen 2004).

## SUMO chains

In the ubiquitin system, different chain connections produce a variety of signals that determine the fate of the modified protein. The SUMOylation includes activation, binding, connection, modification, and dissociation. All SUMO proteins present as immature precursors, requiring the maturation of SUMO proteases to expose the C-terminal diglycine (GG) motif.

SUMOylation affects the activation, interaction, subcellular localization, and stability of substrate proteins. In recent years, a series of new ubiquitin ligases (E3), known as ULS(E3-S) or STUbL, have been discovered that recognize SUMO proteins and associate SUMO modification with the ubiquitin/protease system. SUMO's connection to the substrate also requires a series of enzymatic cascades, including E1 activase (Sae1/Sae2 heterodimer in humans), E2 conjugating enzyme (Ubc9 in humans), and E3 ligase (Guo et al. 2007). Connections between SUMO and other SUMO lysine residues (K) contribute to the formation of substrate connections to the SUMO chain (poly-SUMOylation) (Pfander et al. 2005). SUMO modification is highly dynamic and reversible (Mendler et al. 2016). The SUMO monomer or SUMO chains can be unbound to the protein by the SUMO isopeptidase (ULP, SENP, SUSP). There are three different SUMO proteins in mammals, but only one SUMO homolog in *C. elegans*, named SUMO-1. The homologs of E1 activase in *C. elegans* are AOS-1/UBA-2, E2 conjugating enzyme homologs is UBC-9, and E3 ligase homologs are GEI-17 and MMS-21 respectively.

Like ubiquitin, mature SUMO molecules require activation to modify the substrate, a process performed by the SUMO activating enzyme. SUMO activating enzyme (E1) is a heterodimer formed by AOS1 (SAE1, Sua1) and UBA2 (SAE2), whose N-terminal and C-terminal are similar to the corresponding structures of ubiquitin-activating enzyme (Dohmen et al. 1995). SUMO conjugating enzyme (E2) through transesterification reaction, the activated SUMO molecules are transferred from the SUMO activating enzyme UBA2 subunit to the SUMO conjugating enzyme (E2), also known as UBC9, to form the SUMO-UBC9 sulfur ester intermediate. As one of the main nuclear proteins, UBC9 can locate both the cytoplasmic and cytoplasmic sides of the nuclear pore complex (NPC) (Bossis and Melchior 2006). Several proteins are currently identified as SUMO E3 ligases, and a comprehensive biochemical and structural analysis of three types of proteins (sp-ring (Siz/Pias) family, RanBP2, and ZNF451 family) was performed to provide insight into their SUMO catalytic patterns (Pichler et al. 2002) (Table 1).

## SUMO proteases

The SENP family mediates the de-SUMOylation modification of substrate proteins. Immature SUMO protein has an extended chain of 2–11 amino acids at its C-terminal, and the extended chain needs to be cut off by the SUMO specific protease SENP (sentrin-specific protease), exposing diglycine (Gly-Gly) to become stable form, so as to conduct subsequent modification of the target protein. The human genome encodes six SENPs: SENP1, SENP2, SENP3, SENP5, SENP6, and SENP7(Yeh et al. 2000). Their C-terminal is a conserved protease catalytic domain with about 200 amino acids. However, the N-terminal of different SENPs proteins varies greatly from sequence to structure, which may be related to their ability to precisely act on different target proteins (Garvin et al. 2013; Hang and Dasso 2002; Nishida et al. 2001). In *C. elegans*, the SENP family consists of four SUMO proteases (ubiquitin-like proteases, ULPs) ULP-1, ULP-2, ULP-4, and ULP-5. Ulp-2 has been reported to inhibit E-cadherin and promotes its recruitment to adhesive connections. Moreover, ULP-4 has been reported to deSUMOylate HMGS-1 to control mevalonate pathway activity during aging (Tsur et al. 2015; Sapir et al. 2014).

SENP1 exists in the nuclear pore and nuclear membrane, which can travel between the cytoplasm and the nucleus. SENP2 is the nuclear membrane related protease, which mainly present in the nucleus, but experiments have shown that it is also present in the cytoplasm (Itahana et al. 2006). SENP3 and SENP5, which may be likely to modify SUMO2/3. SENP6 and SENP7 are mainly located in the nucleus (Garvin et al. 2013), which are significantly different from SENP1, SENP2, SENP3, and SENP5 in structure, have an additional ring structure in their catalytic region, mainly in the nuclear material, and de-SUMOylation of SUMO2/3 (Nayak and Muller 2014).

One substrate protein provides more free SUMO protein to modify other substrates as well as de-SUMOylation. The restrictive SUMO precursor, the mature SUMO subtype, and the SENP family determine the SUMO modification balance (Hickey et al. 2012).

## The crosstalk between SUMO modification and apoptosis

**Fig. 1:**
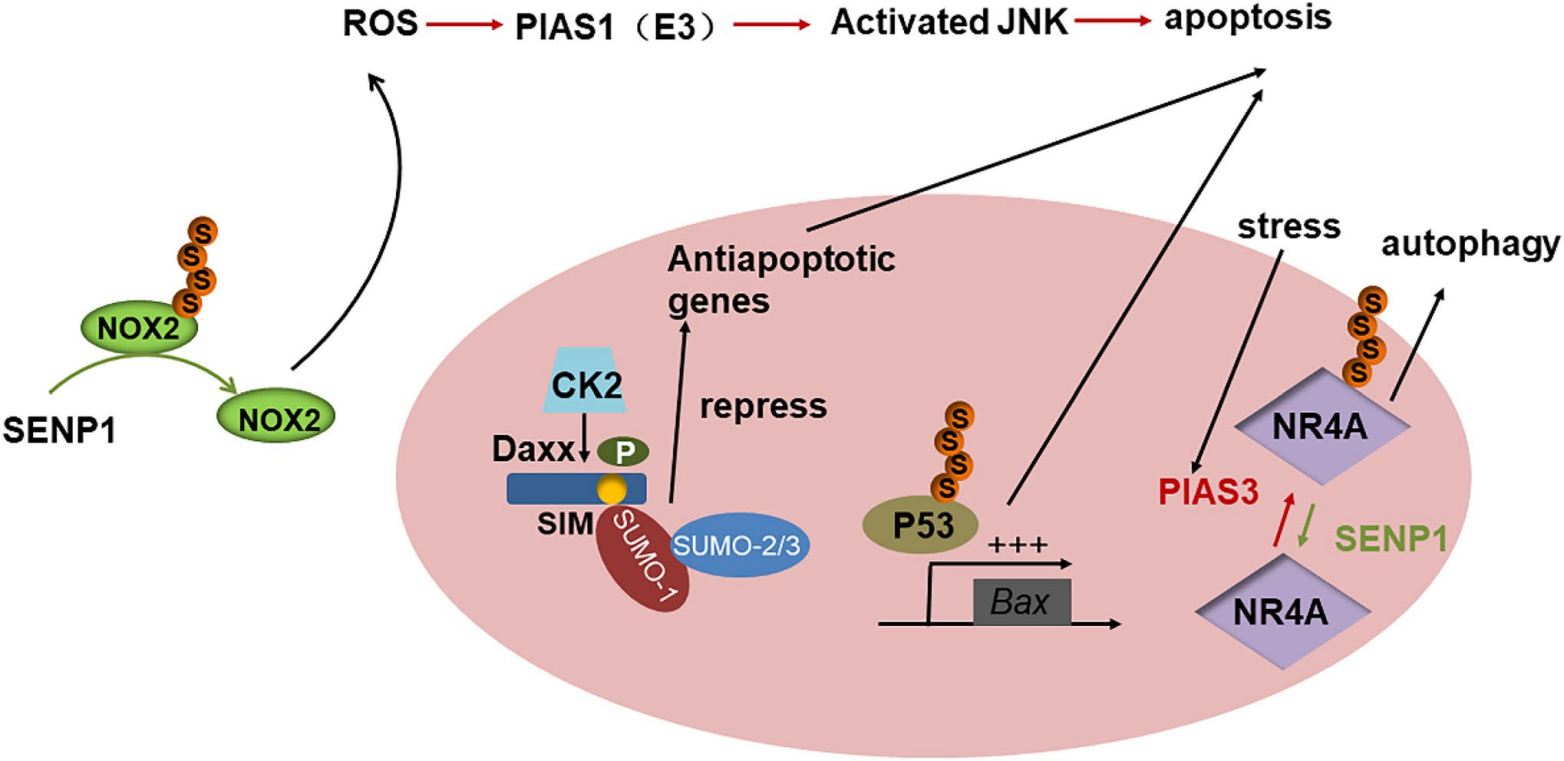
The graphics shows that how SUMOylation regulates apoptosis in cells

SUMO modification is a post-translational modification that affects protein stability, transcriptional activity, protein–protein interactions, and intracellular localization of target proteins. It has been reported that SUMOylation can regulate the activity of NR4A1. NR4A is a nuclear receptor protein family, whose members act as sensors in the cellular environment to regulate metabolism, proliferation, migration, apoptosis, autophagy, and other processes, thus inducing autophagy-dependent cell death. The process is outlined in Fig. 1. Moreover, mutation of three ubiquitin-like sites of NR4A1 can increase its transcriptional activity, alter its intracellular distribution, and more importantly, its ability to induce autophagy death is impaired (Zárraga-Granados et al. 2020; Zhang et al. 2017).

Myeloid cell leukemia 1(MCL1), an anti-apoptotic protein that belongs to the BCL-2 family, often keeps a high expression in many cancers during oncogenesis. The stability of MCL1 is maintained by SUMOylation at its K234 and K238 sites, which inhibits Tripartite motif-containing 11(TRIM11) -mediate ubiquitination of MCL1 and apoptosis of cancer cells (Li et al. 2020).

DAXX(death domain-associated protein) was a kind of FAS binding protein and could act as a regulator of JNK-mediated apoptosis (Baltimore et al. 2000). DAXX has been shown to mediate apoptosis through exogenous death receptor pathways and to regulate gene expression by interacting with multiple DNA-binding transcription factors (TFs) and chromatin related proteins as transcriptional co-inhibitors or co-activators (Hollenbach et al. 2002; Li et al. 2000). In addition, it was reported that DAXX contains SUMO-interacting motifs (SIMs) (Santiago et al. 2009). The Phosphorylation of the Daxx-SIM promotes SUMOylation by binding SUMO-1 to SUMO-2/3, thereby enhances stress-induced cell apoptosis by repressing the antiapoptotic genes (Chang et al. 2011). Some studies have reported that in the case of cell damage caused by reactive oxygen species (ROS), the SUMO ligase PIAS1 can activate the JNK signaling pathway and inhibit the expression of anti-apoptotic genes (Feligioni et al. 2011).

Many recent studies have reported the use of SUMO modification for the treatment of diseases, especially cancer. As the only E2 coupling enzyme in the ubiquitin-like modified pathway, UBC-9 has been widely used in many clinical treatments. Studies have shown that 2-D08, as a small molecular reagent to prevent the process from UBC9 to substrates, may induce the apoptosis of AML cells mediated by ROS accumulation through the de-SUMOylation of NOX2 (Kim et al. 2014; Zhou et al. 2019). In addition, p53, as a tumor suppressor protein, is associated with post-translational modifications of various proteins. When p53 is transferred to the nucleus and modified by SUMO, the transcriptional expression of pro-apoptotic gene *Bax* can be up-regulated (Wasiak et al. 2007). In the rat model of intervertebral disc degeneration (IDD), the positive expression of SUMO2/3 protein was higher. In addition, silencing the SUMO2 gene reduces the expression level and phosphorylation level of p53, while increasing the expression level of CDK2/4 and CyclinB1. Therefore, SUMO2 influences the apoptosis and senescence of nucleus pulposus cells in the IDD rat model by mediating the p53 signaling pathway (Jin et al. 2018).

SUMO modification disorders are likely to result in human diseases. A large number of studies have shown that the SUMO modification is in an unbalanced condition during the development of various cancers (Rabellino et al. 2017). Besides, accelerated apoptosis is involved in neurodegenerative diseases, such as Alzheimer's disease and Parkinson's disease. In addition, SUMO modification protects the body from viruses. However, viral proteins could compete with host cells for the SUMOs to infect bodies. From this perspective, SUMO modification alters virus-host interactions (Zhu et al. 2019; Isaacson and Ploegh 2009).

## Conclusion and prospect

Over the past few decades, we've learned how apoptosis is triggered, what molecules are activated to kill cells, and how phagocytes recognize apoptotic cells. Apoptosis plays an important role in the development of organisms and maintains homeostasis. Timely and effective elimination of apoptotic cells can also prevent the body from producing inflammation or autoimmune diseases due to the release of their harmful contents. Thus, deregulating signaling pathways that trigger cell death can lead to cancer and autoimmune diseases (too little cell death) and degenerative diseases (too much cell death). Certain regulatory pathways can activate or inhibit cell death, depending on the developmental environment. The specific activation of cell death during development need to be further studied, thus help us to understand how cell death is precisely controlled in the treatment of human diseases. By studying the intersection of developmental pathways with conserved core apoptosis mechanisms, we can learn how cancer cells develop resistance to cell death, or how to prevent cell death in neurodegenerative diseases such as Parkinson's disease.

The way a cell dies can have important effects on neighboring cells, and sometimes even the entire organism. The signals that cause death may vary with different types of cell death. The death pathways are clearly interrelated. For example, autophagic death is usually enhanced by caspase activation, which is antagonistic in caspase-dependent necrosis. The interconnections between these pathways may provide several possible mechanisms for cell death procedures (Abraham et al. 2004; Nicotera and Melino 2004).

Over the past 20 years, our understanding of the SUMO mechanism of proteins has expanded greatly. SUMO molecules participate in post-translational modification of proteins, but do not mediate proteasome degradation of target proteins. Instead, they reversibly modify target proteins and participate in their positioning and functional regulation. However, it is not clear how SUMO uses its limited number of enzymes to achieve substrate specificity, compared to a large number of substrates. Moreover, the regulation mechanism of SUMO needs to be further studied.

At present, some progress has been mentioned in the role of SUMOylation in apoptosis. SUMOylation plays an important role in the functional modification of protein substrates. Therefore, elucidating the role of SUMOylation in apoptosis is of great significance for some chronic inflammatory diseases caused by apoptosis and helps us to understand the pathogenesis. However, the current studies on the role of SUMOylation in apoptosis mainly focus on the process of the occurrence of apoptosis, while little is known about the SUMOylation involved in the process of cell corpse clearance. Therefore, the study of SUMOylation in the process of apoptotic cell clearance is helpful for us to further reveal the pathogenesis of chronic inflammatory response caused by apoptotic clearance disorder and provide a new theoretical basis for the development of drugs in clinical practice.
